# Epithelioid inflammatory myofibroblastic sarcoma treated with Alectinib: a case report and literature review

**DOI:** 10.3389/fonc.2024.1412225

**Published:** 2024-08-30

**Authors:** Xinchun Wu, Junxi Zhu, Yichao Yan, Dongfeng Niu, Lin Chen, Ning Ning, Yankai Zhang

**Affiliations:** ^1^ Department of Gastrointestinal Surgery, Peking University International Hospital, Beijing, China; ^2^ Department of Pathology, Peking University Cancer Hospital, Beijing, China

**Keywords:** epithelioid inflammatory myofibroblastic sarcoma, inflammatory myofibroblastic tumor, anaplastic lymphoma kinase, Alectinib, drug allergy

## Abstract

Epithelioid inflammatory myofibroblastic sarcoma (EIMS) is an extremely rare and aggressive form of inflammatory myofibroblastic tumor. Clinically, it has a high risk of relapse and peripheral organ infiltration, and it responds poorly to conventional chemotherapy. Anaplastic lymphoma kinase (ALK) inhibitors are currently the most effective targeted therapy for EIMS. This report discusses a typical case of abdominal EIMS in a 43-year-old woman. The tumors recurred rapidly within one month after surgery. Alectinib was promptly administered upon diagnosis. However, the patient developed a severe allergic reaction to the medication. After a comprehensive assessment and symptomatic treatment, her condition stabilized, leading to a favorable prognosis. This study summarizes cases of abdominal EIMS, highlights the successful use of Alectinib for treatment, and discusses the management of medication-related complications.

## 1 Introduction

Inflammatory myofibroblastic tumor (IMT) is a rare mesenchymal neoplasm with intermediate biological behavior and indolent clinical manifestations (1). It seldom results in distant metastasis and has a relatively low recurrence rate, ranging from 2% to 25% in various studies (2). Histologically, IMTs are primarily composed of proliferating spindle cells and infiltrating lymphocytes. Over 50% of IMTs exhibit rearrangements involving the anaplastic lymphoma kinase (ALK) gene located on chromosome 2p23, leading to abnormal activation of tyrosine kinase and transformation of cell phenotype (3, 4). When tyrosine kinases are activated at the nuclear membrane or in the perinuclear region, the tumor may transform into epithelioid inflammatory myofibroblastic sarcoma (EIMS), which consists mainly of round-to-epithelioid cells and exhibits more aggressive and malignant clinical features (5).

Since Marino et al. (6) first reported EIMS in 2011, dozens of similar cases have been documented over the past 13 years. EIMS has been identified in various organs, including the lung (7), omentum (8), skin (9), and uterus (10). According to existing literature, the recurrence rate after surgery for EIMS is extremely high, and the tumor shows resistance to conventional chemotherapy and immunotherapy. However, several studies have demonstrated that EIMS responds well to ALK inhibitors (6, 8, 12, 13, 15, 20–24, 33, 35). Therefore, we present this case of EIMS treated with the ALK inhibitor Alectinib and review relevant literature to provide guidance for future clinical diagnosis and treatment.

## 2 Case report

**Figure 1:**
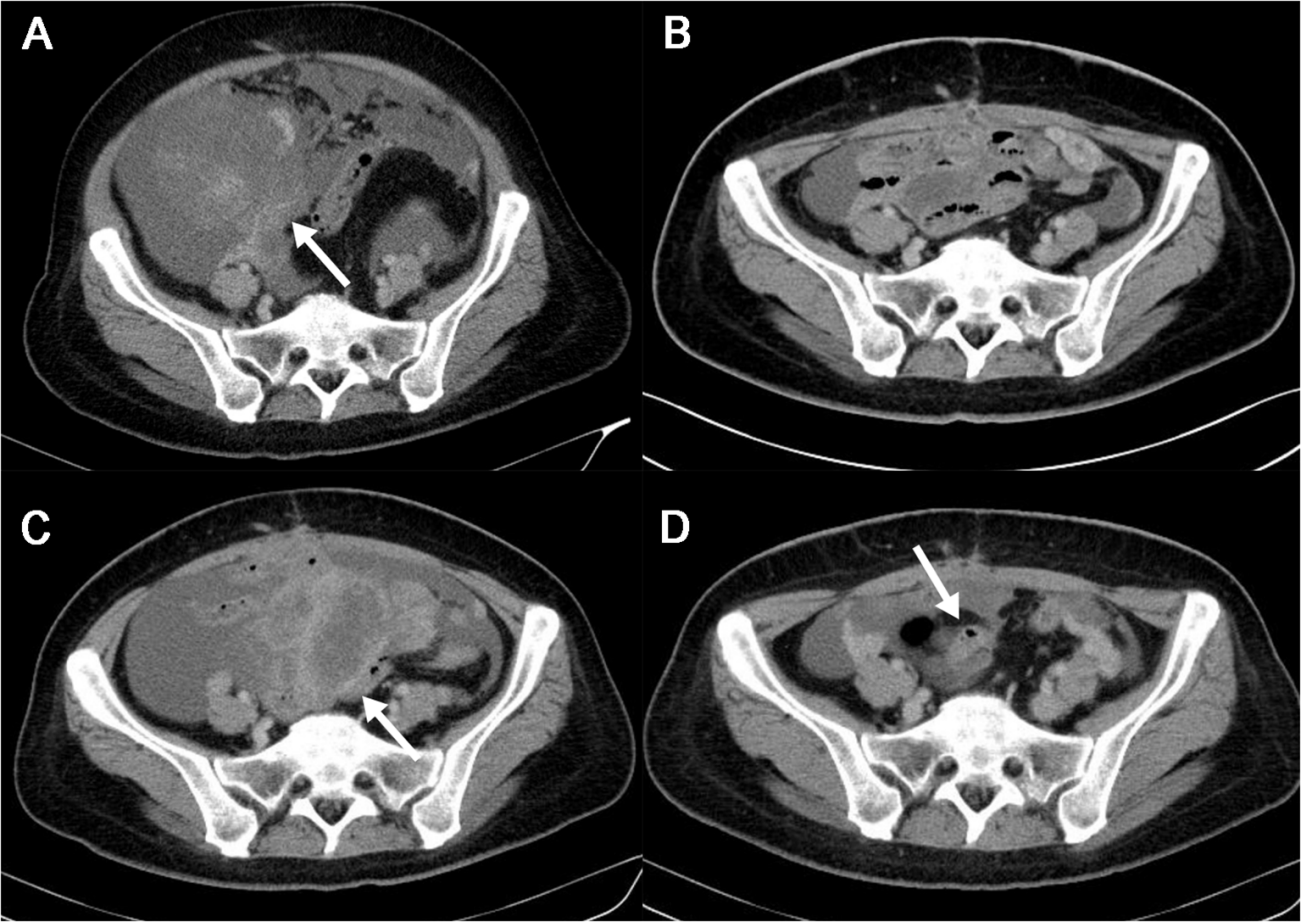
The patient’s imaging features during whole course of the disease in abdominal and pelvic enhanced CT. **(A)**. Preoperative Abdominal and Pelvic Enhanced CT in June, 2023. Multiple soft tissue masses with blurred edge were observed in the abdominal cavity(white arrow). After contrast enhancement, the masses showed obvious uneven enhancement, the largest one was about 19.5*16.8cm. Massive ascites and multiple retroperitoneal enlarged lymph nodes were seen in the abdomen and pelvis. **(B)**. Postoperative Abdominal and Pelvic Enhanced CT in August, 2023. Ascites was still in the pelvic cavity. **(C)**. Abdominal and Pelvic Enhanced CT in September, 2023. Interim assessment of chemotherapy show disease progression(tumors recurred as shown by white arrow). **(D)**. Abdominal and Pelvic Enhanced CT in November, 2023. One month after initiating ALKi treatment, assessment showed significant tumor shrinkage and reduction in ascites(white arrow). ALKi, anaplastic lymphoma kinase inhibitor.

In June 2023, a 43-year-old woman with a history of intermittent abdominal pain since April 2023 presented to our institution. Abdominal and pelvic enhanced CT scans revealed massive ascites and multiple soft tissue density shadows in the abdomen and pelvis. The tumor appeared irregular in shape with ill-defined margins and showed invasion into the bowel (**Figure 1A**). Following a multi-disciplinary team (MDT) discussion, the patient underwent exploratory laparotomy, abdominal mass resection, partial jejunal resection, jejunal anastomosis, ileocecal resection, and ileocolonic anastomosis (postoperative enhanced CT are shown in **s**).

**Figure 2:**
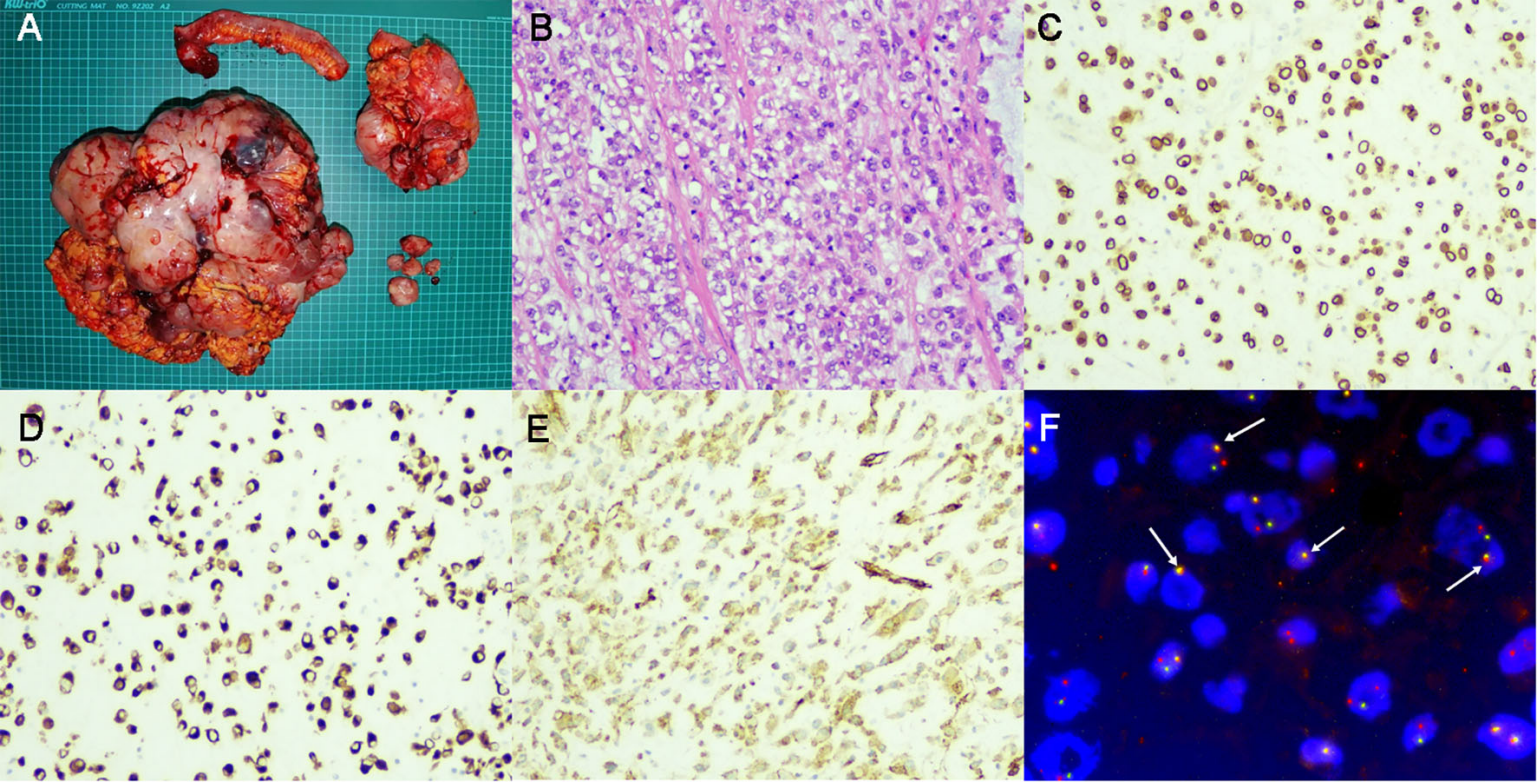
Surgical specimen and pathological examination. **(A)** Gross examination revealed a massive tumor weighing 8kg and adjacent organ involvement. **(B)**. Hematoxylin and Eosin staining showed the existence of both spindle cells and epithelioid cells. **(C)**. IHC staining for ALK revealed positive expression around the nuclei of tumor cells. **(D, E)**. IHC staining showed positive for desmin **(D)** and SMA **(E)**. **(F)**. FISH revealed splitting signals inside tumor cells (As indicated by the white arrow), confirming the presence of ALK rearrangement. IHC, immunohistochemical; ALK, anaplastic lymphoma kinase; FISH, fluorescence *in situ* hybridization.

Postoperative pathology (**Figure 2A**) revealed that the tumors originated from intra-abdominal mesenchymal tissue, with sizes of 35×25×13 cm, 14×11×9 cm, and 5×3×3 cm respectively. The tumor invaded the serosal layer of the intestinal wall to the muscularis mucosae and invaded the muscularis wall of the appendix, assessed as grade 2 in the FNCLCC (Fédération Nationale de Centres de Lutte Contre le Cancer) system. The tumors contained spindle cells and epithelioid cells simultaneously, with lamellar necrosis, mitotic figures of 2-3/10 HPF (high power field), and myxoid stroma (**Figure 2B**). Immunohistochemical (IHC) staining results (**Figures 2C–E**) were as follows: ALK, p16, p53, CDK4, desmin, SMA, S-100, CD34, CD30, CD68, WT1, Calretinin, D2-40, and Ki-67 (approximately 30%) were positive, while CK, MDM2, CD117, DOG-1, Myogenin, and EMA were negative. Based on the IHC results and the microscopic morphology of the tumor, a diagnosis of EIMS was considered.

After the surgery, a Next-generation sequencing (NGS) test was immediately conducted, suggesting potential benefits from 5-fluorouracil, platinum, and PD-1/PD-L1 inhibitors for the patient. Consequently, we opted for the Xelox + sintilimab chemotherapy regimen. Following three cycles of chemotherapy, the patient returned to our institution with recurrent symptoms including abdominal distension, nausea, fever, and a perceived weight gain of 2.5 kg. Imaging examination revealed multiple nodules in the abdomen and pelvis, with sizes up to 2.2×1.7 cm (**Figure 1C**). Chemotherapy assessment indicated progressive disease (PD). Meanwhile, ALK rearrangement was confirmed via fluorescence *in situ* hybridization (FISH) as shown in **Figure 2F**. Based on our literature review and imaging assessments, the patient was initiated on Alectinib treatment (600 mg, BID).

On the 11th day of medication, the patient suddenly developed a high fever, rash, itchy, and other symptoms suggestive of a drug allergy. Following a comprehensive examination, we confirmed the presence of a drug allergy. Referring to the latest expert consensus on the management of adverse drug reactions associated with ALK inhibitors (ALKi) in 2023, we noted that the patient did not exhibit any signs of liver or kidney dysfunction, indicating no contraindication for treatment continuation. Therefore, we initiated symptomatic treatment (details provided in **Table 1**) and decided to proceed with Alectinib therapy. After 18 days of continuous Alectinib therapy, the patient’s abdominal circumference decreased from 100 cm to 92 cm, and all previous complaints resolved. Subsequent abdominal and pelvic enhanced CT scans in November 2023 revealed significant tumor shrinkage and reduction (the maximum tumor diameter decreased from 35.6cm to 5.1cm), along with a notable decrease in ascites (**Figure 1D**). According to the Response Evaluation Criteria in Solid Tumors (RECIST), the patient achieved a partial response (PR) and remained alive with the tumor without experiencing any significant adverse events at the time of manuscript preparation.

**Table 1:** Complications of current case developed during treatment of Alectinib.

Complications	Grade 1*	Grade 2*	Grade 3*	Grade 4*	Grade 5*	Treatment
Dysgeusia	√		_	_	_	Mecobalamin + Vitamin B2
Rash		√		_	_	Olopatadine Hydrochloride + Dexamethasone + Calamine lotion
Itchy			√	_	_	Olopatadine Hydrochloride + Dexamethasone + Calamine lotion
Fever		√				Acetaminophen
Chill	√			_	_	Keep warm
Fatigue		√		_	_	Observe
Headache		√		_	_	Observe
Constipation		√				Increase liquid intake
Anemia			√			Iron supplements
Leukopenia		√			_	Recombinant human granulocyte colony stimulating factor injection

*Grade 1-5 are based on the Common Terminology Criteria for Adverse Events(CTCAE).

“√” means the patients are in the state of adverse event grade corresponding to “√”.

## 3 Discussion

EIMS represents a highly aggressive and malignant variant of IMTs, characterized by epithelioid-to-round cell morphology. Unlike indolent tumors of mesenchymal origin, EIMS typically exhibits invasion into adjacent organs upon discovery by medical teams (3). Consistent with observations made by other researchers, our case demonstrated similar insensitivity to conventional chemotherapy and immunotherapy, posing significant treatment challenges initially. To comprehensively delineate the characteristics of EIMS, we conducted a literature search using PubMed and the China National Knowledge Infrastructure (CNKI), identifying a total of 51 cases of abdominal origin reported in 28 articles, as summarized in **Table 2**.

**Table 2:** EIMS cases originated from abdominal cavity.

Case	Age	Sex	Site	Treatment	Recurrence	Metastasis	Follow up	Reference
1	59y	Male	Mesentery	SE+Che	+	–	12 mo (DOD)	Mariño E, 2011 (6)
2	41y	Male	Omentum	SE+Che+ALKi	+	+	40 mo (ANED)	
3	6y	Male	Omentum	SE+Che	+	–	13 mo (AWD)	
4	28y	Male	Mesentery	NA	NA	NA	NA	
5	63y	Male	Mesentery	SE+Che	+	–	3 mo (DOD)	
6	42y	Male	Mesenchymal tissue	SE+Che	+	–	13 mo (AWD)	
7	7mo	Male	Peritoneum	SE+Che+RT	+	–	36 mo (DOD)	
8	40y	Male	Peritoneum	SE+Che+RT	+	+	28 mo (DOD)	
9	31y	Female	Mesentery	SE+Che	+	–	11 mo (DOD)	
10	6y	Male	Omentum	SE	NA	NA	NA	
11	39y	Male	Mesentery	SE	NA	NA	NA	
12	22y	Male	Mesentery	SE+Che+ALKi	–	+	10 mo (AWD)	Kimbara S, 2014 (12)
13	22y	Male	Ileum	SE+Che+ALKi	+	+	14 mo (AWD)	Kurihara H, 2014 (13)
14	47y	Female	Mesentery	SE+RT	+	–	5 mo (DOD)	Wu H, 2014 (14)
15	22y	Male	Mesentery	SE+ALKi	–	–	16 mo (ANED)	Liu Q, 2015 (15)
16	47y	Female	Mesentery	SE	–	+	5 mo (DOD)	Wu H, 2015 (16)
17	8y	Male	Omentum	SE+Che	+	–	8 mo (DOD)	Zhou J, 2015 (17)
18	38y	Male	Intestine	ST	–	+	8 mo (DOD)	Wanjiao P, 2016 (18)
19	29y	Female	Mesenchymal tissue	SE	–	+	4 mo (DOD)	
20	46y	Male	Omentum	SE	–	+	5 mo (DOD)	
21	46y	Male	Omentum	SE+Che	–	–	6 mo (ANED)	Lijuan Q, 2016 (19)
22	37y	Female	Rectum	SE	–	–	8 mo (AWD)	Yu L, 2016 (20)
23	55y	Male	Ileocecal junction	SE+Che	+	–	10 mo (AWD)	
24	22y	Male	Mesentery	SE+Che+ALKi	+	–	14 mo (AWD)	
25	58y	Female	Abdominal wall	SE+Che	+	–	8 mo (DOD)	
26	15y	Female	Mesentery	SE	–	–	7 mo (AWD)	
27	45y	Male	Mesenchymal tissue	SE+ALKi	+	+	1 mo (DOD)	Jiang Q, 2017 (21)
28	42y	Male	Intra-abdominal	NA	+	–	40 mo (AWD)	Lee JC, 2018 (5)
29	34y	Male	Liver	NA	+	–	5 mo (DOD)	
30	62y	Male	Intra-abdominal	NA	–	–	2 mo (DOD)	
31	76y	Female	Intra-abdominal	NA	–	–	4 mo (DOD)	
32	30y	Male	Intra-abdominal	NA	–	–	8 mo (DOD)	
33	26y	Male	Intra-abdominal	NA	+	+	16 mo (AWD)	
34	39y	Female	Intra-abdominal	NA	+	+	10 mo (AWD)	
35	7mo	Male	Intra-abdominal	NA	–	–	36 mo (DOD)	
36	15y	Female	Ovary	SE+Che+ALKi	+	+	24 mo (AWD)	Fang H, 2018 (22)
37	28y	Male	Mesenchymal tissue	SE+ALKi	+	–	26 mo (AWD)	Xu X, 2019 (23)
38	46y	Female	Mesenchymal tissue	SE+ALKi	+	+	16 mo (DOD)	Zhang S, 2019 (24)
39	35y	Female	Stomach	SE	–	–	13 mo (AWD)	Xu P, 2019 (25)
40	41y	Male	Mesenchymal tissue	ST	–	+	3 mo (DOD)	Yongjie H, 2020 (26)
41	NA	NA	Sigmoid	NA	NA	NA	NA	Liu D, 2020 (27)
42	7y	Male	Stomach	SE	–	–	12 mo (AWD)	Giannaki A, 2020 (28)
43	53y	Male	Descending colon	SE+Che	–	+	7 mo (AWD)	Xiaoyu L, 2021 (29)
44	56y	Female	Mesentery	SE	–	+	5 mo (DOD)	Li D, 2021 (30)
45	4mo	Female	Mesentery	SE	–	–	6 mo (AWD)	Batool S, 2021 (31)
46	43y	Female	Uterus	SE+Che	–	–	22 mo (AWD)	Collins K, 2022 (10)
47	70y	Male	Jejunum	SE	–	–	5 mo (AWD)	Dou W, 2023 (32)
48	20y	Female	Mesenchymal tissue	SE+Che+ALKi	+	–	8 mo (DOD)	Aminimoghaddam S, 2023 (33)
49	31y	Male	Omentum	SE+ALKi	+	–	4 mo (AWD)	Li M, 2023 (8)
50	18y	Female	NA	NA	NA	NA	NA	Fadl A, 2023 (34)
51	26y	Male	Mesenchymal tissue	SE+ALKi	–	–	50 mo (AWD)	Cheng N, 2024 (35)
52	43y	Female	Mesenchymal tissue	SE+Che+ALKi	+	–	9 mo (AWD)	Current case

y, year; mo, month; SE, surgical excision; Che, chemotherapy; RT, radiation therapy; ST, supportive therapy; ALKi, ALK inhibitor; ANED, alive with no evidence of disease; AWD, alive with disease; DOD, dead of disease; NA, data not available.

In summary, EIMS appears to be more prevalent in males (33/52) than females (18/52), with a wide age range of occurrence spanning from 7 months to 70 years. While EIMS can originate from various organs, it is most frequently observed within the abdominal cavity, particularly in the mesentery, omentum, and other mesenchymal tissues (38/52). Three-quarters of patients underwent surgical treatment (39/52), with 27 receiving adjuvant therapy and only 13 receiving ALKi treatment. Among the cases with available prognostic information (approximately 47 cases), 25 experienced postoperative recurrence, while 16 developed postoperative metastasis. Twenty-two patients ultimately succumbed to the disease, whereas 22 remained alive with tumors as documented in their respective articles. Remarkably, only three cases achieved complete recovery without recurrence or metastasis. Notably, among the 13 patients treated with ALKi, only three died, indicating a relatively favorable prognosis for the remaining ten patients.

Regarding IHC staining, our patient exhibited characteristic positivity for ALK, desmin, and SMA. Desmin (36) and SMA (37) are markers of fibroblasts, typically positive in the majority of EIMS cases. ALK positivity in the nuclear membrane or cytoplasm signifies the constitutive activation of ALK, a key factor in the transformation of IMT to EIMS (3, 38). The results of IHC staining for ALK are affected by many factors. For example, different ALK fusion partners can result in diverse expression levels and patterns of the ALK protein, which can affect the staining intensity and distribution observed in IHC staining. Takeuchi’s research found that EML4-ALK fusion showed stronger IHC staining compared to others like KIF5B-ALK or TFG-ALK, highlighting the variability in IHC results based on the fusion partner (39). Besides, the choice of antibody clone used for detecting ALK fusions also plays a crucial role in the sensitivity of IHC. Mino-Kenudson M’s study found the D5F3 clone demonstrated superior performance in detecting a broader range of ALK fusion variants, thus providing more reliable and consistent staining results (40). According to our findings, all cases with complete postoperative pathological information tested positive for ALK, which is quite different compared with the 50% positive rate of ALK in IMTs. This might attribute mainly to the difference of fusion partners between EIMS and IMTs. In addition, among other positive results of IHC staining in our case, p16 (41), p53 (42), CDK4 (43), WT1 (44) are all tumor suppressor genes, their mutation usually indicate strong proliferative activity of tumors. Positive expression of angiogenic marker CD34 (45), macrophage marker CD68 (46), and lymphatic vessel marker D2-40 (47) indicates active formation of tumor interstitial tissue, which usually mean the tumor has strong invasivity. Calretinin (48) and S-100 (49) are both calcium-binding proteins involved in various cellular functions. Their positivity is typically observed in soft tissue tumors. Of note, CD30 is reported positive in over half of cases, suggesting its potential as a diagnostic marker and treatment target. Despite the modest sensitivity and specificity of these markers in existing studies, IHC still holds significant diagnostic value for EIMS before FISH testing becomes available.

In our case, NGS did not detect the presence of genes with ALK rearrangement, which could be due to the detection methods (On the whole exome chip, there are only exon probes for the ALK gene, but the DNA fusion breakpoint is located in the intron). However, literature reports indicate that RANBP2 is the gene most commonly associated with ALK (8, 50). Besides RANBP2-ALK, rearrangement patterns involving RRBP1-ALK (5, 35), TPM3-ALK (5, 51), and EML4-ALK (21) have also been reported in some cases. RANBP1, RANBP2, and RRBP1 are localized to the nuclear membrane and play diverse roles in cell cycle activities, such as nucleocytoplasmic transport, centrosome assembly, microtubule polymerization, spindle assembly, and nuclear envelope remodeling (52–54). This may explain the significant changes in the morphological structure and biological behavior of tumor cells when ALK is rearranged with these genes. Some researchers suggest that RANBP2-ALK rearrangement may lead to ALK expression in the nuclear membrane, while RANBP1-ALK rearrangement may result in ALK expression in the cytoplasm (54–56). Both arrangements contribute to aggressive biological behavior, but further studies are necessary to explore the genetic mechanisms underlying EIMS.

During ALKi therapy, the patient experienced multiple allergic reactions to Alectinib, as outlined in **Table 1**. On October 3rd, 2023, the 11th day following the initiation of ALKi therapy, the patient revisited our institution due to fever, generalized rash, and itching, with a peak body temperature of 39.7°C. Following the diagnosis of drug allergy, immediate treatment was administered based on the Expert Consensus of Management of Adverse Drug Reactions with Anaplastic Lymphoma Kinase Tyrosine Kinase Inhibitors (referred to as “The Consensus” below) (11). Despite the severe fever, discontinuation of the drug wasn’t indicated as there were no signs of liver injury, as emphasized in The Consensus. Additionally, acute anemia was detected during routine laboratory tests on the third day of ALKi therapy, although no significant clinical symptoms were observed. Research indicates that subclinical drug-induced hemolytic anemia and the presence of acanthocytes are common in patients undergoing Alectinib treatment (57, 58). In line with The Consensus, the patient was prescribed iron supplements and regular blood tests. Currently, the patient’s hemoglobin level varies from 110g/L to 120g/L in regular blood tests, without any significant clinical symptoms.

Due to the inefficacy of conventional chemotherapy, there is a growing interest in experimental treatments for EIMS, both in clinical practice and laboratory research. Among these, ALKi has demonstrated promising clinical efficacy, as evidenced by our own clinical observations and literature review. Researchers are also exploring other potential targets. For instance, Li et al. identified a diffuse positive signal for PD-L1 in their EIMS case, suggesting a potential target for PD-1/PD-L1 inhibitors (8). However, initial adjuvant chemotherapy with sintilimab showed no response in our case, despite high PD-1/PD-L1 expression observed in NGS testing. In another study, Fordham AM’s experiments using animal models of allotransplantation revealed that combining a CD30 inhibitor with ALKi resulted in significant tumor shrinkage in both diagnostic and relapse xenograft model groups (59). Additionally, this combination therapy significantly prolonged tumor-free survival in the diagnostic group compared to the relapse group. Based on current experience, the optimal treatment approach for EIMS involves surgical intervention combined with ALKi administered in either adjuvant or neoadjuvant therapy. As more EIMS cases are identified, clinicians will have a broader range of targeted drug options. With ongoing research and a deeper understanding of EIMS pathogenesis, an optimal treatment regimen may gradually emerge, offering patients maximum benefits.

## 4 Conclusion

In conclusion, we presented a detailed case of EIMS characterized by round or epithelioid cell morphology, demonstrating aggressive biological behavior and a poor prognosis. To our knowledge, our report is the first to investigate the efficacy of Alectinib in treating EIMS, while also addressing severe drug-induced complications associated with its administration. Our literature review indicates that ALKi therapy is currently the most effective treatment for EIMS, although other targets may also hold promise. Further studies are warranted to elucidate the pathogenesis of EIMS.

## Data Availability

The original contributions presented in the study are included in the article, further inquiries can be directed to the corresponding author/s.
